# Supplementary material for: vapD Mutation Shows Impairment in the Persistence of Helicobacter pylori Within AGS Cells
Source: Microorganisms. 2025 Aug 21;13(8):1952. doi: 10.3390/microorganisms13081952 (PMC12388325; doi:10.3390/microorganisms13081952)
Supplement: Supplementary file 1 [file microorganisms-13-01952-s001.zip › Figure S1A-B.pdf]

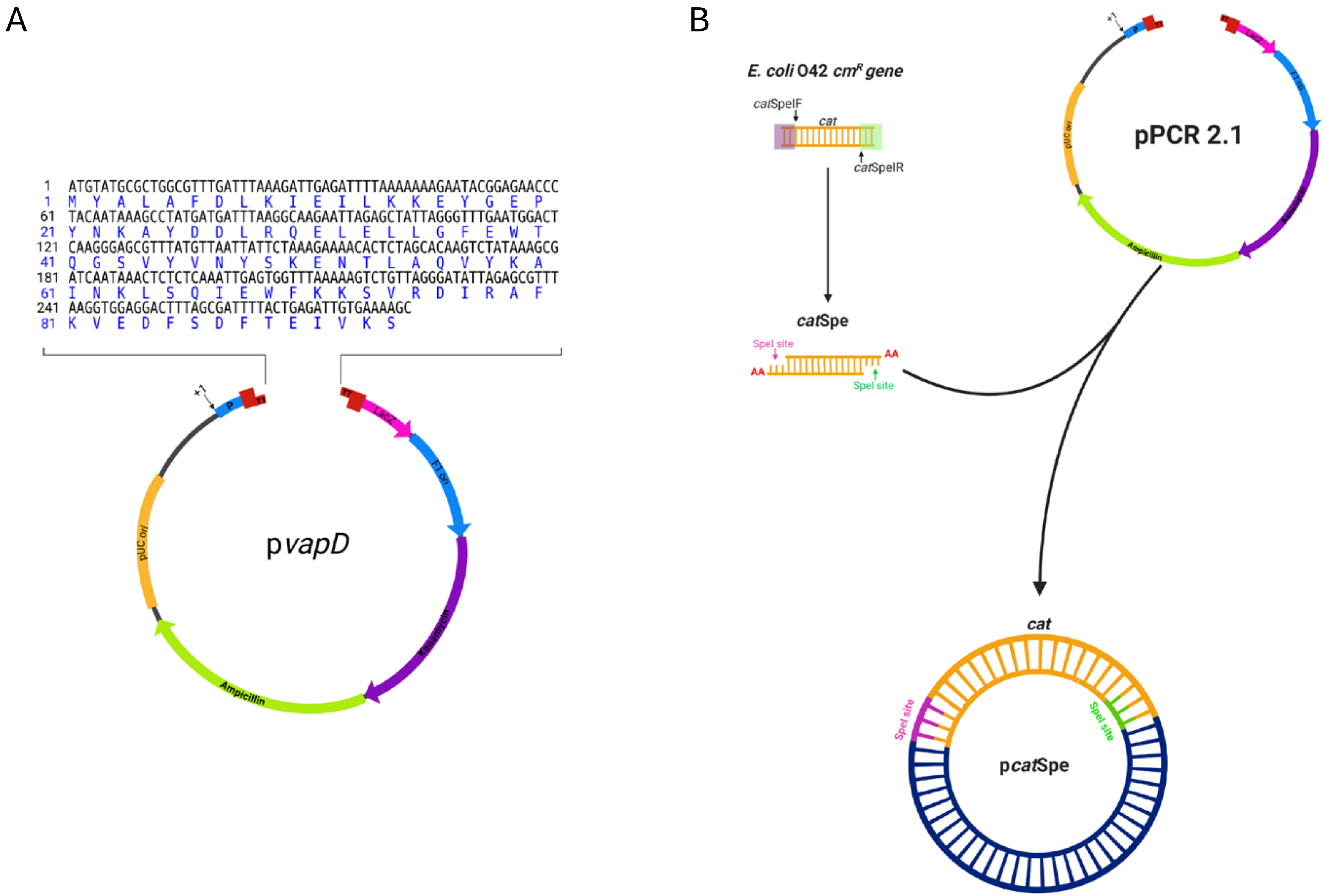

Figure S1A-B. Construction of the *pvapD* and *pcatSpe* plasmids.
