# Supplementary material for: vapD Mutation Shows Impairment in the Persistence of Helicobacter pylori Within AGS Cells
Source: Microorganisms. 2025 Aug 21;13(8):1952. doi: 10.3390/microorganisms13081952 (PMC12388325; doi:10.3390/microorganisms13081952)
Supplement: Supplementary file 1 [file microorganisms-13-01952-s001.zip › Figure S2.pdf]

5’ region of *H. pylori* 26695 ***vapD*** ***cat*** ***vapD*** **3’ region of *H. pylori* 26695**

ACGAGTTAAAAAGAGAAAATAGTAAAGGTTATGTATGCCTAGTAAGAGGTTCCAACCTCAAGGGAGCGTTGTTTAGCCAAATTTTTAA

```
                    10        20        30        40        50        60        70        80        90       100
           ---------+---------+---------+---------+---------+---------+---------+---------+---------+---------+
Hp 26695ΔvapD.seq   ACAGCGAAAAACAGCTTTTTAATTTAATCCATCAATTAGAGCGAAAAATCAAAAAGATGCAAAATGATAGAATTTCTTTTAAAGAAAAAATGGCTAAAGA   100
FUS32R5’.seq        ACAGCGAAAAACAGCTTTTTAATTTAATCCATCAATTAGAGCGAAAAATCAAAAAGATGCAAAATGATAGAATTTCTTTTAAAGAAAAAATGGCTAAAGA   100

                   110       120       130       140       150       160       170       180       190       200
           ---------+---------+---------+---------+---------+---------+---------+---------+---------+---------+
Hp 26695ΔvapD.seq   ATTGGAAAAAAAGGGATCAAAACTTTAAGGATAAAATAGACGCGTTAAATGAACTCTTGCAAAAAATCAGTCAAGCTTTTGATGATAAAAGAGATTGTTGT   200
FUS32R5’.seq        ATTGGAAAAAAAGGGATCAAAACTTTAAGGATAAAATAGACGCGTTAAATGAACTCTTGCAAAAAATCAGTCAAGCTTTTGATGATAAAAGAGATTGTTGT   200

                   210       220       230       240       250       260       270       280       290       300
           ---------+---------+---------+---------+---------+---------+---------+---------+---------+---------+
Hp 26695ΔvapD.seq   TTGGGGCATGAGATCCCAAACATTGAAACGCAACAAGCCATGAGAGATGCGTTAAATGGAATTAATCTCACTCAAATTGATAGTTTAGATGATTTTACAA   300
FUS32R5’.seq        TTGGGGCATGAGATCCCAAACATTGAAACGCAACAAGCCATGAGAGATGCGTTAAATGGAATTAATCTCACTCAAATTGATAGTTTAGATGATTTTACAA   300

                   310       320       330       340       350       360       370       380       390       400
           ---------+---------+---------+---------+---------+---------+---------+---------+---------+---------+
Hp 26695ΔvapD.seq   ACGAGTTAAAAAGAGAAAATAGTAAAGGTTTTGAAAATGTATGCTTTAGCGTTTGATTTAAAGATTGAGATTTTAAAAAAAGAATACGGAGAACCCTACA   400
FUS32R5’.seq        ACGAGTTAAAAAGAGAAAATAGTAAAGGTTTTGAAAATGTATGCTTTAGCGTTTGATTTAAAGATTGAGATTTTAAAAAAAGAATACGGAGAACCCTACA   400
FUS32RM.seq         ---------------------------------------------------------------------------------------------CCCTACA   7

                   410       420       430       440       450       460       470       480       490       500
           ---------+---------+---------+---------+---------+---------+---------+---------+---------+---------+
Hp 26695ΔvapD.seq   ATAAAGCCTATGATGATTTAAGGCAAGAATTAGAGCTATTAGGGTTTGACTAGTAAGAGGTTCCAACTTTCACCATAATGAAATGAGATCACTACCGGGC   500
FUS32R5’.seq        ATAAAGCCTATGATGATTTAAGGCAAGAATTAGAGCTATTAGGGTTTGACTAGTAAGAGGTTCCAACTTTCACCATAATGAAATGAGATCACTACCGGGC   500
FUS32RM.seq         ATAAAGCCTATGATGATTTAAGGCAAGAATTAGAGCTATTAGGGTTTGACTAGTAAGAGGTTCCAACTTTCACCATAATGAAATGAGATCACTACCGGGC   107

                   510       520       530       540       550       560       570       580       590       600
           ---------+---------+---------+---------+---------+---------+---------+---------+---------+---------+
Hp 26695ΔvapD.seq   GTATTTTTTGAGTTATCGAGATTTTCAGGAGCTAAGGAAGCTAAAATGGAGAAAAAAATCACTGGATATACCACCGTTGATATATCCCAATGGCATCGTA   600
FUS32R5’.seq        GTATTTTTTGAGTTATCGAGATTTTCAGGAGCTAAGGAAGCTAAAATGGAGAAAAAAATCACTGGATATACCACCGTTGATATATCCCAATGGCATCGTA   600
FUS32RM.seq         GTATTTTTTGAGTTATCGAGATTTTCAGGAGCTAAGGAAGCTAAAATGGAGAAAAAAATCACTGGATATACCACCGTTGATATATCCCAATGGCATCGTA   207

                   610       620       630       640       650       660       670       680       690       700
           ---------+---------+---------+---------+---------+---------+---------+---------+---------+---------+
Hp 26695ΔvapD.seq   AAGAACATTTTGAGGCATTTCAGTCAGTTGCTCAATGTACCTATAACCAGACCGTTCAGCTGGATATTACGGCCTTTTTAAAGACCGTAAAGAAAAATAA   700
FUS32R5’.seq        AAGAACATTTTGAGGCATTTCAGTCAGTTGCTCAATGTACCTATAACCAGACCGTTCAGCTGGATATTACGGCCTTTTTAAAGACCGTAAAGAAAAATAA   700
FUS32RM.seq         AAGAACATTTTGAGGCATTTCAGTCAGTTGCTCAATGTACCTATAACCAGACCGTTCAGCTGGATATTACGGCCTTTTTAAAGACCGTAAAGAAAAATAA   307

                   710       720       730       740       750       760       770       780       790       800
           ---------+---------+---------+---------+---------+---------+---------+---------+---------+---------+
Hp 26695ΔvapD.seq   GCACAAGTTTTATCCGGCCTTTATTCACATTCTTGCCCGCCTGATGAATGCTCATCCGGAATTCCGTATGGCAATGAAAGACGGTGAGCTGGTGATATGG   800
FUS32R5’.seq        GCACAAG                                                                                              707
FUS32RM.seq         GCACAAGTTTTATCCGGCCTTTATTCACATTCTTGCCCGCCTGATGAATGCTCATCCGGAATTCCGTATGGCAATGAAAGACGGTGAGCTGGTGATATGG   407

                   810       820       830       840       850       860       870       880       890       900
           ---------+---------+---------+---------+---------+---------+---------+---------+---------+---------+
Hp 26695ΔvapD.seq   GATAGTGTTCACCCTTGTTACACCGTTTTCCATGAGCAAACTGAAACGTTTTCATCGCTCTGGAGTGAATACCACGACGATTTCCGGCAGTTTCTACACA   900
FUS32RM.seq         GATAGTGTTCACCCTTGTTACACCGTTTTCCATGAGCAAACTGAAACGTTTTCATCGCTCTGGAGTGAATACCACGACGATTTCCGGCAGTTTCTACACA   507

                   910       920       930       940       950       960       970       980       990      1000
           ---------+---------+---------+---------+---------+---------+---------+---------+---------+---------+
Hp 26695ΔvapD.seq   TATATTCGCAAGATGTGGCGTGTTACGGTGAAAACCTGGCCTATTTCCCTAAAGGGTTTATTGAGAATATGTTTTTCGTCTCAGCCAATCCCTGGGTGAG   1000
FUS32RM.seq         TATATTCGCAAGATGTGGCGTGTTACGGTGAAAACCTGGCCTATTTCCCTAAAGGGTTTATTGAGAATATGTTTTTCGTCTCAGCCAATCCCTGGGTGAG   607

                  1010      1020      1030      1040      1050      1060      1070      1080      1090      1100
           ---------+---------+---------+---------+---------+---------+---------+---------+---------+---------+
Hp 26695ΔvapD.seq   TTTCACCAGTTTTGATTTAAACGTGGCCAATATGGACAACTTCTTCGCCCCCGTTTTCACCATGGGCAAATATTATACGCAAGGCGACAAGGTGCTGATG   1100
FUS32RM.seq         TTTCACCAGTTTTGATTTAAACGTGGCCAATATGGACAACTTCTTCGCCCCCGTTTTCACCATGGGCAAATATTATACGCAAGGCGACAAGGTGCTGATG   707
FUS32R3’.seq        ----------------------------------------------------------------------------------------ACAAGGTGCTGATG   14

                  1110      1120      1130      1140      1150      1160      1170      1180      1190      1200
           ---------+---------+---------+---------+---------+---------+---------+---------+---------+---------+
Hp 26695ΔvapD.seq   CCGCTGGCGATTCAGGTTCATCATGCCGTTTGTGATGGCTTCCATGTCGGCAGAATGCTTAATGAATTACAACAGTACTGCGATGAGTGGCAGGGCGGGG   1200
FUS32RM.seq         CC                                                                                                   709
FUS32R3’.seq        CCGCTGGCGATTCAGGTTCATCATGCCGTTTGTGATGGCTTCCATGTCGGCAGAATGCTTAATGAATTACAACAGTACTGCGATGAGTGGCAGGGCGGGG   114

                  1210      1220      1230      1240      1250      1260      1270      1280      1290      1300
           ---------+---------+---------+---------+---------+---------+---------+---------+---------+---------+
Hp 26695ΔvapD.seq   CGTAATTTTTTTAAGGCAGTTATTGGTGCCCTTAAACGCCTGGTTGCTACGTCTGAATAAGTGATAATAACTAGTCTCAAGGGAGCGTTTATGTTAATTA   1300
FUS32R3’.seq        CGTAATTTTTTTAAGGCAGTTATTGGTGCCCTTAAACGCCTGGTTGCTACGTCTGAATAAGTGATAATAACTAGTCTCAAGGGAGCGTTTATGTTAATTA   214

                  1310      1320      1330      1340      1350      1360      1370      1380      1390      1400
           ---------+---------+---------+---------+---------+---------+---------+---------+---------+---------+
Hp 26695ΔvapD.seq   TTCTAAAGGAAACACTCTAGCACAAGTCTATAAAGCGATCAATAAACTCTCTCAAATTGAGTGGTTTAAGAAGTCTGTTAGGGATATTAGAGCGTTTAAG   1400
FUS32R3’.seq        TTCTAAAGGAAACACTCTAGCACAAGTCTATAAAGCGATCAATAAACTCTCTCAAATTGAGTGGTTTAAGAAGTCTGTTAGGGATATTAGAGCGTTTAAG   314

                  1410      1420      1430      1440      1450      1460      1470      1480      1490      1500
           ---------+---------+---------+---------+---------+---------+---------+---------+---------+---------+
Hp 26695ΔvapD.seq   GTGGAGGACTTTAGCGATTTTACTGAGATTGTGAAATCCTAGTGTTTAGCCAAATTTTTAAAATTTTTGGCACTTATAGAGTGGAAAGCAACGCTTTTTT   1500
FUS32R3’.seq        GTGGAGGACTTTAGCGATTTTACTGAGATTGTGAAATCCTAGTGTTTAGCCAAATTTTTAAAATTTTTGGCACTTATAGAGTGGAAAGCAACGCTTTTTT   414

                  1510      1520      1530      1540      1550      1560      1570      1580      1590      1600
           ---------+---------+---------+---------+---------+---------+---------+---------+---------+---------+
Hp 26695ΔvapD.seq   AGTAAGACTAGGCAGTCATAGCGAGCTGTTTTGTGAAAACTACCCATAACGCTTAAAAAAAAAATGCAACAATCGCATGTTAGCCCTAAACCCCCTATTAA   1600
FUS32R3’.seq        AGTAAGACTAGGCAGTCATAGCGAGCTGTTTTGTGAAAACTACCCATAACGCTTAAAAAAAAAATGCAACAATCGCATGTTAGCCCTAAACCCCCTATTAA   514

                  1610      1620      1630      1640      1650      1660      1670      1680      1690      1700
           ---------+---------+---------+---------+---------+---------+---------+---------+---------+---------+
Hp 26695ΔvapD.seq   AAAATAACTTAAGCGTTCAACTCTATTTTTTGCGATGCTCTTTTTAAGCCTAGCCCATAGTTAAATCTCTGTTTCATCAATTGATCACCACCGCAAAAAA   1700
FUS32R3’.seq        AAAATAACTTAAGCGTTCAACTCTATTTTTTGCGATGCTCTTTTTAAGCCTAGCCCATAGTTAAATCTCTGTTTCATCAATTGATCACCACCGCAAAAAA   614

                  1710      1720      1730      1740      1750      1760      1770      1780      1790      1800
           ---------+---------+---------+---------+---------+---------+---------+---------+---------+---------+
Hp 26695ΔvapD.seq   GCCTTATTCTAGTAAAAGCCTTGTTTTTATGAAGCGCTTTAGACACAAGCCCTGTAACGCTAATAACCAACTCTTTTTCATTCAATCGTGGGG          1793
FUS32R3’.seq        GCCTTATTCTAGTAAAAGCCTTGTTTTTATGAAGCGCTTTAGACACAAGCCCTGTAACGCTAATAACCAACTCTTTTTCATTCAATCGTGGGG          707
```

Figure S2. Complete nucleotides sequence of the recombinant region (FUS32) of *vapD* knockout (Hp26695ΔvapD.seq).
F32R5’corresponds to the nucleotide sequences (1 bp-708 bp) amplified by PCR with FUS32R5’F/R primers.
F32RM corresponds to the nucleotide sequences (394 bp-1102 bp) amplified by PCR with FUS32RM’F/R primers.
F32R3’ corresponds to the nucleotide sequences (1085 bp-1793 bp) amplified by PCR with FUS32R3’F/R primers.
The color of the nucleotide sequences was preserved in relation to those used in the figure 1.
