# Supplementary material for: vapD Mutation Shows Impairment in the Persistence of Helicobacter pylori Within AGS Cells
Source: Microorganisms. 2025 Aug 21;13(8):1952. doi: 10.3390/microorganisms13081952 (PMC12388325; doi:10.3390/microorganisms13081952)
Supplement: Supplementary file 1 [file microorganisms-13-01952-s001.zip › Table S1.pdf]

Table S1. Protocol conditions used of each PCR for the construction of knockout vapD::Cm^r.

PCR	Primer set	PCR conditions	MW
vapD::Cm^r	1F / 1R	94°C for 1 min — 1cycle<br>94°C for 30 s<br>Tm for 30 s } 30 cycles<br>68°C for 2 min<br>68°C for 5 min — 1cycle	285bp
PCR3/PCR2	—	95°C for 2 min — 1cycle<br>95°C for 1 min<br>68°C for 10 s<br>66°C for 5 s } 20 cycles<br>64°C for 5 s<br>62°C for 5 s<br>68°C for 10 min — 1cycle	—
FUS1	5F/6R	94°C for 2 min — 1cycle<br>94°C for 30 s<br>52°C for 30 s } 30 cycles<br>68°C for 3 min<br>68°C for 10 min — 1cycle	~2.9 Kb
PCR2/PCR4 (FUS4)	*cat*SpeIF/7*sal*R	94°C for 2 min — 1cycle<br>94°C for 30 s<br>52°C for 30 s } 30 cycles<br>68°C for 3 min<br>68°C for 10 min — 1cycle	~2.6 Kb
FUS1/FUS4	—	95°C for 2 min — 1cycle<br>95°C for 1 min } 30 cycles<br>68°C for 4 min<br>68°C for 10 min — 1cycle	—
FUS32	FUS32F/FUS32	94°C for 2 min — 1cycle<br>94°C for 30 s<br>61°C for 30 s } 30 cycles<br>68°C for 3 min<br>68°C for 10 min — 1cycle	~3.6 Kb
